# Editorial: DNA virus and host plant interactions from antagonism to endogenization

**DOI:** 10.3389/fpls.2022.1014516

**Published:** 2022-09-08

**Authors:** Katja R. Richert-Pöggeler, Marie-Line Iskra-Caruana, Yuji Kishima

**Affiliations:** ^1^Julius Kuehn Institute, Federal Research Centre for Cultivated Plants, Institute for Epidemiology and Pathogen Diagnostics, Braunschweig, Germany; ^2^CIRAD, DGD-RS, Montpellier, France; ^3^Laboratory of Plant Breeding, Research Faculty of Agriculture, Hokkaido University, Sapporo, Japan

**Keywords:** plant DNA viruses, virus evolution, virus integration, RNAi, virus ecology

The findings and reviews in the article collection of the Research Topic on “*DNA Virus and Host Plant Interactions from Antagonism to Endogenization*” investigate the complexity of parasitic, mutual, or commensal virus interactions with the host cell and highlights the evolved diversity of DNA viruses infecting major crop plants, ornamentals as well as weeds.

Even though by current definition viruses are not regarded as living organisms, their regulative force within the ecosystem and their impact on evolution of life mediating horizontal DNA transfer is more and more revealed and recognized (Suttle, 2007; Gilbert and Feschotte, 2018; Loiseau et al., 2021). Significant knowledge has been obtained from studies focusing on viruses of bacteria, animals and humans (Krupovic and Forterre, 2015; Pisano et al., 2020) but less is known for the environment comprising plants and particular for their infecting DNA virus(es). This is surprising since almost 3 decades ago, the literally “breakthrough” of viral sequences into the plant genome had been established for geminiviruses (Bejarano et al., 1996) as well as for some pararetroviruses, taxon *Caulimoviridae*, neither of them relying on chromosomal integration in their replication cycle (Hohn et al., 2008).

Plant DNA viruses belong either to the families of *Geminiviridae* or *Nanoviridae* harboring single stranded (ss)DNA genomes in their capsids or to the family *Caulimoviridae* that encapsidate double stranded (ds)DNA genomes. Viruses being acellular, parasitic entities occur generally as episomes in their host cell on which their replication depends (Figure 1).

**Figure 1:**
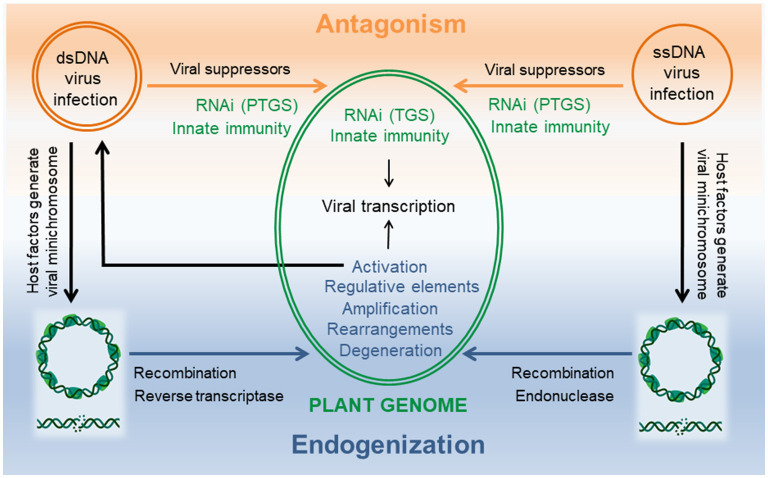
DNA viruses and host plant interactions from antagonism to endogenization. Successful viral infection is established when viral suppressors can overcome the host cellular defense machinery consisting of RNAi and innate immunity. Multiple levels of interactions in the nucleus as well as in the cytoplasm are possible during this arms race. Common to both viruses is a viral minichromosome that is generated using host factors and serves as template for viral transcription in the nucleus. Double strand DNA breaks result in linearized molecules that can promote endogenization by illegitimate recombination and/or interference with reverse trancriptase (dsDNA viruses) or endonuclease (ssDNA viruses). Evolution of the chimeric host-virus genome can create diversity by rearrangements, insertion of viral regulative elements, amplification and/or degeneration of virus sequences. Integrants that allow transcription of a genome length RNA are the source for virus replication and systemic infection. Minichromosomes and dsDNA breaks created with BioRender.com.

For survival, DNA viruses that compete with their host for DNA synthesis had to adapt to cellular niches that allow their multiplication without interfering with the host DNA replication in the nucleus. Thus, plant DNA viruses have evolved different strategies. They copy the host's nuclear DNA structures by forming viral minichromosomes and employ for their transcription the host plant DNA-dependent RNA polymerase II (Hull, 2014). *Geminiviridae* synthesize their genomic ssDNA using rolling-circle replication in the nucleus, whereas *Caulimoviridae* generate the genomic dsDNA using reverse transcription in the cytoplasm.

When genotoxic agents damage the viral episomes, they change their topology from circular to linearized molecules. These can be recognized by the host DNA repair machinery leading to multiple pathways of genome invasions by illegitimate recombination (Richert-Pöggeler et al.). Additionally, for geminiviruses the multifunctional replication initiator protein (Rep) encoded by ORF AC1 comprising also endonuclease activity is likely to play a key role in promoting host genome accessions of viral sequences (Hanley-Bowdoin et al., 2013; Ruhel and Chakraborty, 2019). It is noteworthy, that in *Macademia* viral sequences originating from both types of DNA viruses have been reported (Zakeel et al., 2021).

In order to infect their hosts successfully, viruses have evolved various approaches to overcome the RNAi based plant surveillance system in the nucleus as well as in the cytoplasm as illustrated and discussed by Richert-Pöggeler et al. and Zhai et al. In case of Croton yellow vein mosaic virus, a monopartite begomovirus, and its cognate beta-satellite four viral suppressors of RNAi (VSR), namely V2, C2, C4, and βC1 are developed to overcome plant defense mechanism and establish a sustainable infection. Furthermore, the authors reveal distinct functions of the investigated viral suppressors according to their subcellular localization, interactions and roles in symptom induction and intercellular movement.

The importance of miRNAs regulating both host as well as viral gene expression for plant immunity is illustrated by beet curly top virus interactions with its host sugar beet (Majumdar et al.). The observed cross-kingdom RNAi, e.g., plant derived miRNAs targeting the viral capsid protein supports the hypothesis of a chimeric scenario for the origin of viruses, which postulates that replicons existed at an precellular stage and proteins for virion formation derived from the host (Krupovic et al., 2019).

The assembled publications of this Research Topic give examples for the dynamics in autonomous DNA virus evolution as well as in co-evolution with their hosts during endogenization. Thus generated viral diversity and spectrum of virus-host interferences require adapted detection methodology as well as risk assessments (Silva et al.; Umber et al.). This is especially true for collections of germplasm from major food crops like yam and banana. The optimized multiplex PCR-dependent denaturing gradient gel electrophoresis facilitated the screening significantly and allowed comprehensive detection and analysis of endogenous Dioscorea bacilliform viruses (Silva et al.). Umber et al. revealed the dynamics of activation for infectious endogenous pararetroviruses (EPRVs) in banana. The authors paid special attention in their risk studies to cultivation methods comparing tissue culture with field cultivation and indicated the impact of time and altitude respectively for activation on EPRVs in banana.

Design of bioinformatics pipelines are seminal for exploration and functional analyses of integrated viral DNA sequences and related transcriptomes in the plant hosts (Serfraz et al.). Such comprehensive approach identified two previously unreported endogenous badnaviruses in the genus *Solanum*. In depth analysis of the genomic location of these endogenous badnaviruses found them adjacent or within the late blight resistance gene of their host *Solanum melongena*. Moreover, the authors located Ty-1 copia mobile elements–also known as *Pseudoviridae*- in this genomic niche. Future studies addressing the mechanisms that resulted in the co-localization of reverse transcribing elements such as *Caulimoviridae* and phylogenetically closely related *Metaviridae* as well as *Pseudoviridae* are highly desirable.

The ongoing global warming creates selection pressure on evolution of viruses, their associated vectors and hosts resulting in adaptation to the new environment (Amari et al., 2021). Thereby triggered changes in virus epidemiology, host range and pathogenicity can contribute to the emergence of novel viral diseases (Elena et al., 2014).

It will take the united efforts of virologists covering all taxonomic kingdoms to understand and preserve virosphere as well as to predict virus emergence and to prevent future virus outbreaks.

## Author contributions

All authors listed have made a substantial, direct, and intellectual contribution to the work and approved it for publication.

## Funding

YK was supported by the Japan Society for the Promotion of Science program Grants-in-Aid for Scientific Research (JSPS KAKENHI No. 19H00937).

## Conflict of interest

The authors declare that the research was conducted in the absence of any commercial or financial relationships that could be construed as a potential conflict of interest.

## Publisher's note

All claims expressed in this article are solely those of the authors and do not necessarily represent those of their affiliated organizations, or those of the publisher, the editors and the reviewers. Any product that may be evaluated in this article, or claim that may be made by its manufacturer, is not guaranteed or endorsed by the publisher.
